# A typology of healthcare pathways after hospital discharge for adults with COVID-19: the evolution of UK services during pandemic conditions

**DOI:** 10.1183/23120541.00565-2022

**Published:** 2023-08-14

**Authors:** Linzy Houchen-Wolloff, Charlotte Overton, Andrew Ibbetson, Archie Walters, Claire Hastie, Rhyan Gill, Natalie Armstrong, Sally Singh, Paul Little, Kirby Evans, John Pimm, Michael Marks, Krisnah Poinasamy, Sam Walker, Andrew Briggs, Rachael A. Evans

**Affiliations:** 1 Leicester NIHR Biomedical Research Centre – Respiratory, University Hospitals of Leicester NHS Trust, Leicester, UK; 2 Department of Respiratory Sciences, University of Leicester, Leicester, UK; 3 SAPPHIRE, Department of Population Health Sciences, College of Life Sciences, University of Leicester, Leicester, UK; 4 Department of Health Services Research and Policy, London School of Hygiene and Tropical Medicine, London, UK; 5 Long COVID Support, Birmingham, UK; 6 Leicester NIHR Biomedical Research Centre- Respiratory Patient and Public Involvement Group, University Hospitals of Leicester NHS Trust, Leicester, UK; 7 Department of Medicine, University of Southampton, Southampton, UK; 8 Oxford Health NHS Foundation Trust, Oxford, UK; 9 Department of Clinical Research, London School of Hygiene and Tropical Medicine, London, UK; 10 Hospital for Tropical Diseases, University College Hospital London, London, UK; 11 Division of Infection and Immunity, University College London, London, UK; 12 Asthma and Lung UK, London, UK

## Abstract

**Introduction:**

Over half of post-COVID-hospitalisation adults have persistent symptoms 2 years after discharge, providing a challenge for individuals and healthcare systems. We therefore aimed to describe a typology of UK healthcare pathways post-hospital discharge as a first step towards understanding clinical effectiveness and cost-effectiveness of different healthcare pathways.

**Methods:**

In 2021, we surveyed hospital sites taking part in the UK Post-hospital COVID-19 (PHOSP-COVID) study. The online survey explored the availability of proactive follow-up, patient selection, involvement of multidisciplinary teams, investigations, assessment and access to mental health and rehabilitation interventions. The typology was defined by a three-stage process: 1) using the survey results to develop a bespoke algorithm to inform a draft classification, 2) a stakeholder event for refinement and 3) finalisation between the Project Advisory Group and research team. The bespoke algorithm was used to map each site onto the classification with further mapping by level of mental health and rehabilitation provision.

**Results:**

70% of hospital sites (45 out of 64) responded to the survey. 82% (37 out of 45) reported delivering a follow-up service after hospital discharge during the first few months of the pandemic. Only 13 out of 37 services (35%) were delivered by permanent staff. The final typology of five categories included no proactive follow-up, and a matrix of four groups based on patient selection (prespecified subgroup/all patients) and complexity of assessment (low/high). The complexity of assessment, rehabilitation and mental health interventions was variable within sites.

**Discussion:**

We describe the first typology of post-hospitalisation COVID-19 healthcare pathways to enable modelling of clinical effectiveness and cost-effectiveness to inform future policy. Our results highlight the heterogeneity and vulnerability of healthcare services after COVID-19 hospitalisation.

## Tweetable abstract

**A novel classification of post-COVID-19 services involving patient selection, assessment and intervention highlights the heterogeneity of healthcare offered for this population and is the first step to evaluate cost-effectiveness** https://bit.ly/3UcCSz0

## Introduction

At the time of writing, since its first description over 2 years ago, the novel coronavirus SARS-CoV-2 has infected at least 750 million people worldwide and resulted in over 6.5 million deaths [1]. At the start of the pandemic, the severity of the acute illness and the need for inpatient hospital care was dominated by acute lung injury [2]. The long-term outcomes for survivors of COVID-19 were unknown, but lessons learned from other pandemics (such as the SARS 2002–2004 pandemic) indicated that some survivors would have complications of pulmonary fibrosis and post-viral syndromes [3], in addition to physical and mental health deficits, and therefore would require follow-up within a healthcare pathway. A “care pathway” is defined as “a complex intervention for the mutual decision-making and organisation of care processes for a well-defined group of patients during a well-defined period” [4].

In the UK and similar to the international experience, during the first wave of the COVID-19 pandemic (February to September 2020) individual hospital teams made their own judgements about what follow-up they would provide, and to which patients. The first specific UK guidance for respiratory follow-up was published in May 2020 [5]. Long COVID clinics were funded in England from November 2020 and the service specification to date [6] uses available National Institute for Health and Care Excellence (NICE) guidance [7] and expert opinion in the absence of research informing clinical effectiveness and cost-effectiveness [8]. Similarly, a recent narrative scoping review including 37 international studies reported that the delivery of post-COVID rehabilitation is currently based on expert opinion [9]. To date there is minimal published data regarding the establishment of post-COVID/long COVID services internationally [10], though several models in the USA have been described [11]. It is therefore currently unclear how to optimally implement and stratify follow-up services to be both clinically effective and cost-effective. Learning from the COVID pandemic could inform future healthcare pathways for other respiratory infections and the resulting multisystem effects.

We therefore aimed to describe the range of healthcare pathways implemented for patients discharged from hospital after COVID-19 in the UK and ultimately to design a classification of healthcare pathways to be used to assess the clinical effectiveness and cost-effectiveness of care models in future studies.

## Methods

Healthcare pathways were evaluated from centres taking part in the UK Post-Hospitalisation (PHOSP-COVID) study (study registration ISRCTN10980107; Yorkshire & The Humber -Leeds West Research Ethics Committee REC ref: 20/YH/0225). Initial findings from this study have previously been reported [12]. At the time of the current study, the PHOSP-COVID study involved 64 hospital sites across the four nations of the UK. Data were collected *via* an online survey (Joint Information Systems Committee (JISC) online surveys) developed by the study team (which included health economists, a cardiorespiratory physiotherapist, a respiratory physician, a psychologist, a general practitioner, an infectious diseases consultant, a qualitative researcher, patients and a patient and public involvement lead) and based on clinical experience of post-COVID syndrome and the NICE long COVID guidance [7]. The survey focused on the assessment and interventions available for a patient post-discharge after a hospital admission with COVID-19. These included clinical assessment, investigations, follow-up and interventions such as recovery/rehabilitation programmes and specific mental health interventions. For example, chest X-ray was a recommended follow-up investigation for those admitted with COVID pneumonitis and therefore access to this was captured by the survey [5]. Models of delivery were recorded (*e*.*g*. face to face, telephone or video conference), as well as details of how clinics were structured, particularly whether there was a COVID multidisciplinary team meeting and which professionals were part of this. Questions were developed to understand which professionals delivered the key components in the pathway and their employment status. Information on which patients had access to services was collected, including any stratification or eligibility criteria based on patient characteristics, results of investigations or severity of acute illness (such as receiving mechanical ventilation).

The survey consisted of single and multiple choice and, where necessary, free text questions. Prior to rollout a pilot of the survey was sent to two PHOSP-COVID sites for feedback and adaptations made (final survey in the supplement 1). The final survey was sent *via* email to the Principal Investigator (PI) for each of the 64 individual PHOSP-COVID study sites (49 trusts/health boards). The PI was predominately the lead clinician of the post-COVID clinic; they could complete the survey themselves or forward it to the most appropriate person to complete (*e.g.* the clinical lead for the service where this was not the PHOSP PI or rehabilitation lead for the relevant questions). To aid completion, three follow-up emails were sent to non-responders and a telephone call was offered to discuss survey answers if required.

### Data extraction and analysis

Data were exported and categorical data described as proportions and percentages within Excel (Microsoft Office 2010, Microsoft, Redmond, WA, USA). The survey responses for each individual site were checked *via* JISC to corroborate the whole Excel dataset. For multiple choice selections, more than one answer could be provided and therefore the percentage of responses for some questions was >100%.

### Classification of post-hospital healthcare pathways

A three-stage process was employed to define the classification of post-hospital healthcare pathways. First, we reviewed the survey data at a series of meetings with the study team and Project Advisory Group (PAG) to draft an initial classification of post-hospitalisation care pathways. Second, we held an online stakeholder event using Zoom (Zoom Video Communications, Inc. San Jose, CA, USA) to refine the initial classification of pathways. For this we used stakeholder methodology [13] and involved representatives of key stakeholders including service users with lived-experience COVID-19 (COVID-19 managed both in the community and hospital, n=3), clinical/academic collaborators within the PHOSP-COVID working groups including mental health and rehabilitation healthcare professionals (n=6), clinical leads of COVID follow-up services (n=10) and healthcare policy makers (n=7). An agenda for the stakeholder event is shown in supplement 2: table S1. Discussion among the stakeholders was facilitated by two of the authors (LH-W, CO) and focused on the core components to be included, and how to categorise each of the core components. Third, the revised classification was taken to the PAG for further discussion and comment before being finalised by the authors.

For the assessment service mapping, a post COVID-19 Health Service Assessment Algorithm was developed. Further mapping of services by level of rehabilitation and mental health and psychological services provision was overseen by experts in the field. This involved checking through the individual survey responses per site, including the free text responses. A final classification for rehabilitation and mental health services was agreed. Throughout the process, the study team was aware that the final classification should be simple enough that it could inform the health economic analysis of the parent project, of which the research reported here was one workstream. The aim of the broader project was to assess the effectiveness, cost and cost-effectiveness of the different post-hospitalisation care packages adopted across the UK.

### Patient and public involvement

This work orginated from a joint patient and clinician research priority setting exercise [14]. Patient and public representatives helped develop the original proposal and reviewed the survey before the pilot distribution phase. Patient and public involvement tasks included reviewing the mapping survey and the subsequent typology of services within the consensus event and PAG meetings (three patient members attending the PAG).

## Results

We conducted a survey of 64 sites between 23 April and 6 September 2021. The number of sites responding to the survey was 45 out of 64 (70%). Of the 45 sites that responded, 32 sites (71%) were in England, six (13%) in Wales, six (13%) in Scotland and one (2%) in Northern Ireland. Of respondents, 33 (73%) reported delivering a follow-up service after hospital discharge in wave one and 37 (82%) in wave two. The proportion of sites in each country offering a follow-up service at wave two was 29 out of 32 (91%) in England, three out of six (50%) in Wales, four out of six (67%) in Scotland and one of one (100%) in Northern Ireland.

Services were commissioned (the process by which health and care services are planned, purchased and monitored) at 10% of sites in wave one and 25% of sites in wave two. In wave one, only 33% of sites had staff working in post-COVID clinics in permanent positions. This only increased to 35% in wave two. Supplement 2: table S2 outlines the nature of staffing in the two waves, which was predominately redeployed/temporary staffing.

The main changes reported in the provision of services between waves one and two were an 11% increase in centres with a follow-up clinic (33 to 37 sites), an 18% increase in sites having a designated clinical lead (28 to 33 sites) and a 42% reduction in those following-up all patients routinely (12 to 7 sites, *i.e.* sites were using some stratification to decide which patients to follow-up). Hereafter, the data are presented for wave two only because the two waves were similar and this represents the most up-to-date information.

### Structure and content of access and assessment

**FIGURE 1:**
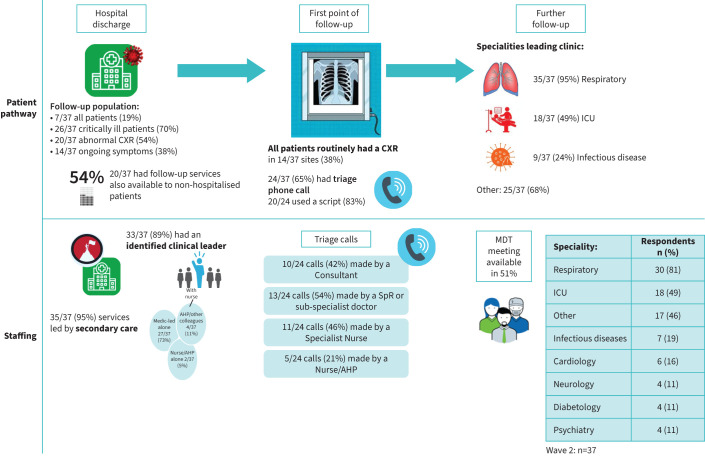
Infographic detailing patient access and assessment across the sites. CXR: chest X-ray; ICU: intensive care unit; MDT: multidisciplinary team; AHP: Allied Health Professional; SpR: specialist registrar.

An overall infographic of the healthcare pathways is shown in figure 1.

#### Clinical leadership and staffing

Overall, 35 out of 37 follow-up services (95%) were delivered in secondary care (typically hospital outpatient clinics) and 33 out of 37 services (89%) had a designated clinical service lead (*i.e.* a clinician leading and responsible for the service, ideally with allocated time to do this (figure 1)). The clinical lead was most commonly a consultant physician alone (27 out of 37; 73%) or in combination with an allied healthcare professional (AHP) or nurse (2 out of 37; 5%), a clinical fellow (1 out of 37; 3%) or other consultants (1 out of 37: 3%). Two out of 37 services (5%) had either an AHP or nurse as the sole lead. The staffing, however, was predominantly temporary with only 13 out of 37 services (35%) delivered by permanent staff (supplement 2: table S2). Redeployed/temporary staff was the most utilised group across sites to manage the follow-up clinics (18 out of 37; 49%).

#### Patient selection/access

By wave two, the majority of sites did not follow up every patient routinely and most sites (30 out of 37; 81%) employed stratification for selecting patients for follow-up (figure 1).

#### Structure of the services

For the majority of sites (24 out of 37; 65%), a triage telephone assessment was used as the first contact and to decide which patients needed further interventions or follow-up. The telephone call was the only follow-up for 11 out of 37 sites (30%). A wide range of staff disciplines conducted the triage telephone call (figure 1).

#### Investigations

The proportion of sites requesting a chest radiograph for all discharged patients with COVID-19 was 14 out of 37 (38%) (figure 1). There was limited access to basic investigations at the assessment (supplement 2: table S3), with only 23 out of 37 sites having access to chest X-ray, phlebotomy and lung function testing.

#### Clinic follow-up

The majority of services had respiratory professionals leading the consultations (35 out of 37; 95%) with intensive care professionals (18 out of 37; 49%) being the second most common specialty (figure 1 and supplement 2: table S4). In terms of meetings, 19 out of 37 sites (51%) had access to a COVID-specific multidisciplinary team meeting to discuss patients, including physicians, nurses, physiotherapists and a range of clinical specialties (supplement 2: table S5).

### Interventions

#### Rehabilitation

**FIGURE 2:**
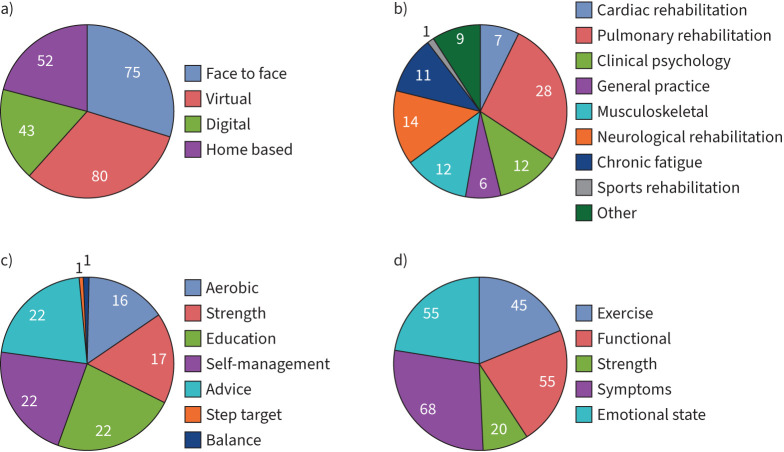
a) Pie charts of rehabilitation models, b) services delivering rehabilitation, c) components of rehabilitation delivered and d) outcomes assessed. Numbers represent the percentage of responses.

Of the 45 sites who responded to the survey, 34 (76%) reported having access to a form of rehabilitation and of these half were COVID-specific programmes. 22 out of 34 sites (65%) with access to rehabilitation completed the survey questions specific to rehabilitation (supplement 1). Most programmes were delivered by the same organisation providing the post-hospitalisation assessment. Figure 2 outlines the details of delivery for rehabilitation models (figure 2a), services delivering rehabilitation (figure 2b), components of rehabilitation delivered (figure 2c) and outcomes assessed (figure 2d). Models included face to face, virtual, digital platforms (*e.g.* www.yourcovidrecovery.nhs.uk) and home-based models. 19 out of 22 sites (86%) used more than one model of delivery. Decisions on how to deliver the programme depended upon available staff, service pressures, safety and patient preference. Programmes were delivered most often by AHPs and existing Pulmonary Rehabilitation services (16 out of 22; 73%); three out of 22 (14%) were managed by a single profession. Not all programmes matched outcome measures to the components delivered (*e.g.* the programme delivered exercise, yet an exercise outcome measure was not assessed at baseline or follow-up).

#### Psychology and mental health services

**FIGURE 3:**
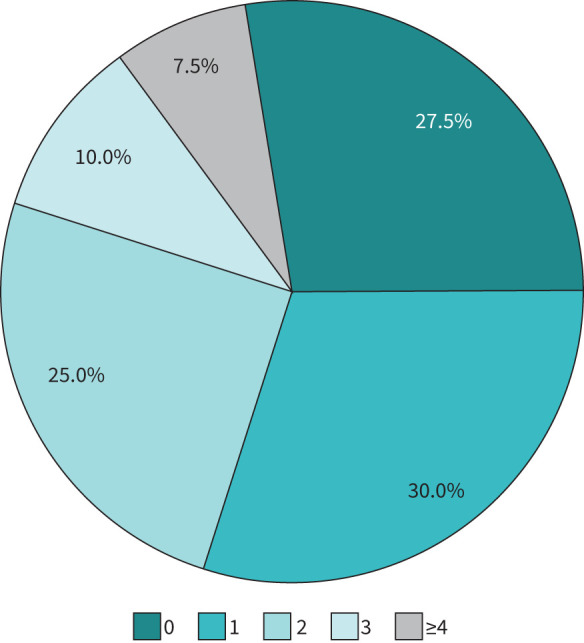
The number of mental health services identified as available by post-COVID clinics.

Table 1 outlines the range of psychological/mental health services that patients had access to in wave two. Over a quarter of centres (10 out of 37; 27%) did not identify any mental health services they could access (figure 3).

**TABLE 1:** Access to the range of mental health/psychological services in wave two.

**Service**	**Respondents with access to the service** **n (%)**
**IAPT**	15 (41)
**Post ICU psychology service**	13 (35)
**Psychiatric liaison service**	7 (19)
**Acute hospital clinical health psychology service**	5 (14)
**Community mental health team (adult)**	4 (11)
**Counselling services (third sector or primary care based)**	4 (11)
**Other**	3 (8)
**Community mental health team (older adult)**	2 (6)
**Mental health crisis resolution and home treatment service**	2 (6)
**Inpatient mental health service**	2 (6)
**Psychological therapies service for serious mental illness**	1 (3)
**Private providers of psychological therapy**	0 (0)

Data provided for wave two (n=37). IAPT: Improving Access to Psychological Therapies; ICU: intensive care unit.

### Results of the three-stage process to develop the typology of healthcare pathways

For the first stage, a set of algorithm questions (supplement 2: figure S1) was developed to create a draft classification of post-hospitalisation care pathways using the survey results. The questions were applied in the following order:

- Did patients have access to a COVID-19 post-hospitalisation follow-up service?
  o If no, were COVID-19 patients followed up in other services?
  o If yes, what were the follow-up services?
- Were all discharged patients invited to attend clinic?
  o If no, was patient attendance stratified?
  o If yes, how were patients stratified?
- Which other clinicians were available in the post-hospitalisation service?
- Did the service include access to a COVID multidisciplinary team meeting?

**FIGURE 4:**
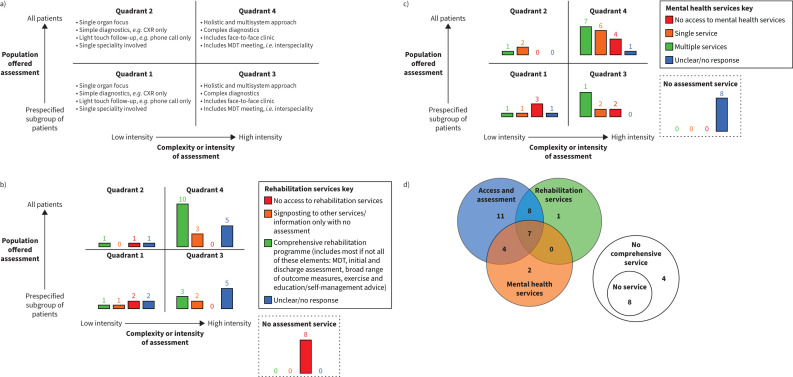
a) Classification matrix for health service assessment. Quadrant 1: low intensity and prespecified subgroup of patients (n=6 sites); Quadrant 2: low intensity and all patients (n=3 sites); Quadrant 3: high intensity and prespecified subgroup of patients (n=10 sites); Quadrant 4: high intensity and all patients (n=18 sites). The prespecified subgroups relates to the decision-making from individual sites, *e.g.* some sites only followed up those who had been in intensive care or who had abnormal chest imaging. b) Number of sites by level of assessment and level of rehabilitation intervention. c) Number of sites by level of assessment and level of mental health service provision. d) A Euler diagram to highlight the overlap between comprehensive/high intensity follow-up services for COVID-19 across the metrics of access and assessment, rehabilitation and mental health services. CXR: chest X-ray; MDT: multidisciplinary team.

The draft classification, produced by the study team in consultation with the PAG, was further developed in the second stage at a stakeholder event (supplement 2: figure S2). For the third stage, the final classification after further discussion with the PAG and research team is shown in figure 4a. Assessment was categorised across two-dimensions: 1) The population offered assessment, either all patients or a prespecified subgroup of patients (*e.g.* some centres only followed up those patients who had required intensive care or had abnormal chest imaging); 2) the complexity or intensity of assessment broadly based upon whether follow-up was holistic and multisystem or a focused follow-up (*e.g.* respiratory only). Together with “no service” this gave five categories that could be taken forward to examine the effectiveness and cost-effectiveness of healthcare pathways. Although the classification system could have been made more granular, there was a concern that doing so would pose problems for the subsequent health economic analysis by generating too many pathways for effective evaluation.

Rehabilitation services were classified as comprehensive/multidimensional, unidimensional, no access to rehabilitation or missing/no response. A comprehensive/multidimensional service was defined as including multidisciplinary teams, initial and discharge assessments either side of the programme, a broad range of outcome measures (*e.g.* symptoms, exercise capacity, quality of life, psychological health) and exercise plus education. Services addressing only one clinical problem, *e.g.* speech and language therapy, were classified as unidimensional.

Mental health services were classified as no services identified as available, single service identified, multiple services and unclear or no response. Figure 3 outlines the number of mental health services available per post-COVID clinic.

Figure 4 provides a full summary of hospital sites by assessment and level of rehabilitation (figure 4b) and mental health service provision (figure 4c), respectively. Although there was some alignment between the level of interventions provided (rehabilitation or specific mental health) and the level of clinical assessment, in some sites this was not closely matched. We therefore did not integrate the level of intervention available into the 2×2 matrix. To embed the mental health and rehabilitation services within the overall categorisation would involve a third and potentially fourth dimension, which may be too granular for the planned health economic model.

An alternative representation is given in figure 4d, which presents a Euler diagram to show the overlap between the complexity/intensity of follow-up services for COVID-19 across the metrics of patient selection, assessment and rehabilitation and mental health services.

## Discussion

We completed a survey in April–September 2021 of post-hospital healthcare pathways for survivors of COVID-19 from the start of the pandemic. Our main results show the heterogeneity of healthcare offered to this population, from no proactive follow-up (*i.e*. discharge home and patient to contact general practitioner as needed) to all patients receiving at least a telephone assessment after discharge. We synthesised our survey results using a novel bespoke algorithm to develop the first classification of post-hospitalisation COVID-19 healthcare pathways based on patient access to the services and the level of assessment offered. Mental health and rehabilitation interventions were mapped onto the classification but there was not a consistent relationship between the complexity of services offered.

In the UK by 30 September 2022, 993 757 patients had been admitted to hospital with COVID-19; internationally, admissions due to COVID-19 continue, although at a reduced rate [15]. In one large UK cohort study, only one in four people felt fully recovered at 1-year post-discharge [12] and in a cohort from Wuhan, China, over half of patients had persistent symptoms 2 years after discharge [16]. Both cohort studies reported a large range of symptoms and mental, physical and cognitive health impairments, highlighting the need for a proactive multisystem approach, a holistic assessment and for interventions to improve post-COVID sequelae. An efficient but good-quality service is therefore essential for patients and the healthcare system.

In our survey, the follow-up services were mainly delivered by the hospitals and predominantly by respiratory healthcare professionals. Over half of sites with a follow-up service had a multidisciplinary team meeting with different specialists and disciplines, reflecting the multisystem involvement post-COVID-19. Most services used a telephone call for the first assessment, and chest radiography was a common investigation performed in line with the British Thoracic Society guidelines (May 2020) for post-COVID follow-up [5]. A striking feature was the vulnerability of services, many without formal commissioning (payer) and nearly half delivered by staff in temporary/redeployed positions. Perhaps surprisingly there were few changes from wave one to wave two. There were recognised tensions in the health systems during this time because healthcare professionals had to balance the rapid transformation of services, volume of acute care, redeployment and routine non-COVID work, as well as their own health and managing staff sickness [17].

Our results are similar to a recently published survey of European post-COVID services [10] where 80% of respondents were from University Teaching Hospitals, 82% had multidisciplinary team involvement and 57% included both hospitalised and non-hospitalised patients within their services. Furthermore, most centres in this survey similarly included some form of stratification for inclusion. The healthcare pathways described from our survey were set up before any national guidance for post-COVID services was available. Further related work describes the situated, structural and systemic resilience related to the development of post-COVID follow-up healthcare pathways [18]. In December 2020, NICE issued rapid guidance for managing the long-term effects of COVID-19 [7]. This guidance includes a comprehensive clinical assessment and appropriate examination that involves assessing physical, cognitive, psychological and psychiatric symptoms, as well as functional abilities, and onward referral to rehabilitation and psychological services as appropriate.

Our survey has shown that rehabilitation was offered in the majority of sites surveyed (76%) and usually delivered by the same secondary care centre. This is in contrast to the results of an international scoping review, where rehabilitation services were integrated at all levels of a health system from primary care to tertiary hospital-based care [9]. We found that only 15 of 34 sites (44%) offered comprehensive rehabilitation (exercise and education supervised by a multidisciplinary team). In 75% of cases, this was offered as a face-to-face programme. However digital/telerehabilitation is an emerging intervention that has been considered as a suitable alternative to traditional centre-based services in those with long-term conditions to deliver accessible, cost-effective and efficient rehabilitation services when patient/programme factors hinder a face-to-face visit [19]. Often these interventions were delivered by existing services rather than developing bespoke COVID programmes. Pulmonary rehabilitation teams were commonly involved in delivering post-COVID rehabilitation. Pulmonary rehabilitation, a programme of exercise and education typically delivered to those with chronic lung disease, was identified as a potentially suitable service for patients with COVID-19, particularly for those struggling with breathlessness [20], by a joint international consensus statement [21]. The current literature identifies that while pulmonary rehabilitation may form a firm foundation, there needs to be a more comprehensive service to manage the breadth of symptoms reported [22]. COVID-specific rehabilitation programmes have thus been implemented online and face to face with promising clinical outcomes [23, 24]. However, there are concerns that COVID-specific rehabilitation may “exacerbate the fragmentation of services” and take priority over other long-term conditions [22].

A wide range of specific mental health services were available according to the results of our survey, but there was heterogeneity between sites. The mapping exercise for mental health services indicates that increasing clinic complexity is likely to be associated with better access to a range of mental health services. Specifically, low-complexity clinics have a higher proportion of no access to mental health services, and high-complexity clinics have a higher proportion of access to multiple mental health services (figure 4c). Post-COVID clinics were most likely to identify access to Improving Access to Psychological Therapies programmes (where available in England) and acute hospital psychological services rather than specialist community mental health services.

The level of access and complexity of the rehabilitation and mental health interventions did not fully match the level of access and complexity of the assessment provided. In terms of the proportions able to access rehabilitation and mental health services, our findings are in keeping with the results of a Europe-wide survey (approximately three quarters with access to both interventions in both surveys [9]). We add new knowledge by demonstrating no clear association with level of assessment and rehabilitation provision. However, there was an association between the complexity of the follow-up services and the range of mental health services they identified. The lack of relationship between the complexities of services offered may also highlight silo working within localities between the different services. The large number of adults with long COVID (needing rehabilitation and psychological support) coupled with the backlog remaining from the crisis is likely to challenge existing services for the foreseeable future [22]. The burden of long COVID extends to non-hospitalised patients recovering from COVID-19. The minimal research published to date has focused on describing post-COVID pathways [11] and rehabilitation access [9]; however, the need to understand both clinical effectiveness and cost-effectiveness of follow-up services is a timely priority.

Anecdotally, patient advocacy groups describe a “postcode lottery” for the post-COVID services on offer [25]. Patients describe the inherent uncertainty within long COVID and the fear and helplessness that people experienced as a result, particularly regarding whether recovery from the condition was even possible [26, 27]. The experience of navigating care pathways post-COVID has been described as “unacceptable” and “exhausting” [27, 28]. There is a particular area of concern around exacerbating health inequalities that have already been highlighted by the pandemic [29] when the default post-COVID service is a reactive one (*i.e.* where patients have to seek help for ongoing symptoms rather than being proactively assessed). The planned analysis to investigate the clinical effectiveness, cost and cost-effectiveness of the different healthcare pathways may shed some light on the relationship between apparent “complexity” and the effectiveness of service provision and provides an opportunity to level-up, standardising the pathway for patients in the future to ensure acceptability, safety and equitable access.

Our survey has covered a range of different hospitals across the four nations of the UK, but the majority were academic teaching hospitals and may not be representative of all UK hospitals who admit patients with acute COVID-19. We did not conduct a formal Delphi process to develop the classification but rather we used a stakeholder event including experts in the field, multiple people with lived-experience of long COVID and post-COVID sequelae, and policymakers [13]. A further limitation was that with survey-level data we were unable to make assumptions about statistical significance between the classifications.

## Conclusion

Owing to the ongoing volume of patients admitted to hospital internationally, coupled with the lack of recovery at 2 years, we urgently need to understand the optimal healthcare pathway for patients and healthcare systems. We have developed a novel classification of post-COVID services to be used to evaluate the clinical and cost-effectiveness of healthcare pathways after a hospital admission for COVID-19.

## Supplementary material

10.1183/23120541.00565-2022.Supp1 **Please note:** supplementary material is not edited by the Editorial Office, and is uploaded as it has been supplied by the author.
PHOSP Mapping Service Survey 00565-2022.SUPPLEMENT
Supplementary tables and figures 00565-2022.supplementary_tables
