# Supplementary material for: A typology of healthcare pathways after hospital discharge for adults with COVID-19: the evolution of UK services during pandemic conditions
Source: ERJ Open Res. 2023 Aug 14;9(4):00565-2022. doi: 10.1183/23120541.00565-2022 (PMC10423987; doi:10.1183/23120541.00565-2022)
Supplement: Supplementary file 1 [file 00565-2022.SUPPLEMENT.pdf]

PHOSP Mapping Service Survey

Post-Hospitalisation COVID-19 Follow-up Service Mapping Survey

Introduction

Post-Hospitalisation COVID-19 Follow-up Service Mapping Survey

The aim of the Post-hospitalisation COVID-19 study (PHOSP) is to understand and improve long-term outcomes for survivors of a hospitalisation with COVID-19. The study includes a Health Service Research component, which aims to understand the impact, facilitators and barriers to implementing holistic post discharge follow-up clinics in the context of COVID-19. This research has three components:

1. Mapping the post-hospital care pathway provision at the NHS Trusts participating in the national study through a survey.
2. Interviews with healthcare staff and managers at selected case study sites.
3. Interviews with patients (and their carers) who have engaged with the care pathways at the case study sites.

Initially, to establish what post-discharge services are in place we are making contact with all of the sites who are taking part in PHOSP study to request completion of this survey.

You may need assistance of the post-hospitalisation COVID-19 service lead and rehabilitation team to complete the questions.

Please note: only one person can complete the survey at a time.

If you have any questions please contact Dr Charlotte Overton, Health Service Researcher, via email - charlotte.overton@leicester.ac.uk

Service Mapping Questions

1. What is the name of the hospital you work in?

2. What is your role the in the post-hospitalisation COVID-19 service?

3. In wave one (Feb-Sept 2020) did patients have access to a COVID-19 post hospitalisation follow-up service (e.g. triage phone call or clinic)?

- Yes
- No

3.a. If no, were COVID-19 post-hospitalised patients followed-up in other services?

- Yes
- No

3.a.i. If yes, what were the follow-up services?

4. In wave two (Oct 2020-March 2021) did patients have access to a COVID-19 post hospitalisation follow-up service (e.g. triage phone call or clinic)?

- Yes
- No

4.a. If no, were COVID-19 post-hospitalised patients followed-up in other services?

- Yes
- No

4.a.i. If yes, what were the follow-up services?

5. In wave one (Feb-Sept 2020) was there a lead clinician for the COVID-19 post hospitalisation follow-up service?

- Yes
- No

5.a. If yes, what is their profession?

- ☐ Doctor
- ☐ Nurse
- ☐ AHP
- ☐ Other

5.a.i. If you selected Other, please specify:

6. In wave two (Oct-2020-March 2021) was there a lead clinician for the COVID-19 post hospitalisation follow-up service?

○ Yes ○ No

6.a. If yes, what is their profession?

- ☐ Doctor
- ☐ Nurse
- ☐ AHP
- ☐ Other

6.a.i. If you selected Other, please specify:

7. In wave one (Feb-Sept 2020) was the post hospitalisation follow-up service commissioned?

○ Yes ○ No

8. In wave two (Oct 2020-March 2021) was the post hospitalisation follow-up service commissioned?

○ Yes ○ No

9. In wave one (Feb-Sept 2020) how was post hospitalisation follow-up service staffed? Please select all that apply.

- ☐ Redeployed staff
- ☐ Permanent staff
- ☐ Temporary staff
- ☐ Other

9.a. If you selected Other, please specify:

10. In wave two (Oct 2020-March 2021) how was post hospitalisation follow-up service staffed? Please select all that apply.

- [ ] Redeployed staff
- [ ] Permanent staff
- [ ] Temporary staff
- [ ] Other

10.a. If you selected Other, please specify:

11. What was the start date for the service for patients discharged from hospital?

Dates need to be in the format 'DD/MM/YYYY', for example 27/03/1980.

(dd/mm/yyyy)

12. Is the service available for patients whose acute illness was managed in the community?

( ) Yes ( ) No

12.a. If yes, what was the start date for this service?

Dates need to be in the format 'DD/MM/YYYY', for example 27/03/1980.

(dd/mm/yyyy)

13. In wave one (Feb-Sept 2020) were all discharged patients invited to attend the clinic?

( ) Yes ( ) No

13.a. If no, was patient attendance stratifed?

( ) Yes ( ) No

13.a.i. If yes, please select how patients were stratified.

- ☐ Patients who required intensive/high dependency care.
- ☐ Patients who required high levels of oxygen and/or CPAP
- ☐ Symptomatic patients
- ☐ Abnormal follow-up chest x-ray
- ☐ Other

13.a.i.a. If you selected Other, please specify:

14. In wave two (Oct 2020-March 2021) were all discharged patients invited to attend the clinic?

○ Yes ○ No

14.a. If no, was patient attendance stratifed?

○ Yes ○ No

14.a.i. If yes, please select how patients were stratified.

- ☐ Patients who required intensive/high dependency care
- ☐ Patients who required high levels of oxygen and/or CPAP
- ☐ Symptomatic patients
- ☐ Abnormal follow-up chest x-ray
- ☐ Other

14.a.i.a. If you selected Other, please specify:

15. In wave one (Feb-Sept 2020) was patient attendance at the clinic decided following a triage phone call?

○ Yes ○ No

15.a. If yes, which staff group made the triage call? Please select all that apply.

- ☐ Consultant
- ☐ Registrar
- ☐ Staff Grade
- ☐ Sub-registrar

- [ ] Medical student
- [ ] Specialist Nurse (please tick other and state speciality)
- [ ] Nurse (please tick other and state grade)
- [ ] Physiotherapist
- [ ] Other

15.a.i. If you selected Other, please specify staff group:

15.b. Was a script/proforma used in the triage phone call?

○ Yes ○ No

16. In wave two (Oct 2020-March 2021) was patient attendance at the clinic decided following a triage phone call?

○ Yes ○ No

16.a. If yes, which staff group made the triage call? Please select all that apply.

- [ ] Consultant
- [ ] Registrar
- [ ] Staff Grade
- [ ] Sub-registrar
- [ ] Medical student
- [ ] Specialist Nurse (please tick other and state speciality)
- [ ] Nurse (please tick other and state grade)
- [ ] Physiotherapist
- [ ] Other

16.a.i. If you selected Other, please specify staff group:

16.b. Was a script/proforma used in the triage phone call?

○ Yes ○ No

17. In wave one (Feb-Sept 2020) which patients received a follow-up chest x-ray? Please select all that apply.

- [ ] All patients

- ☐ Patients who required intensive/high dependency care.
- ☐ Patients who required high levels of oxygen and/or CPAP
- ☐ Symptomatic patients
- ☐ Other

17.a. If you selected Other, please specify:

18. In wave two (Oct 2020-March 2021) which patients received a follow-up chest x-ray? Please select all that apply.

- ○ All patients
- ○ Patients who required intensive/high dependency care.
- ○ Patients who required high levels of oxygen and/or CPAP
- ○ Symptomatic patients
- ○ Other

18.a. If you selected Other, please specify:

We would now like to understand more about the structure and content of post-hospitalisation services in wave one and wave two.

19. In wave one (Feb-Sept 2020) which clinician led the initial consult? Select as appropriate.

	Consultant	Registrar	Staff Grade	Specialist Nurse	Advanced Clinical Practitioner	Physiotherapist	Clinical Psychologists	Occupational Therapists	Dieticians	Speech and Language Therapist
Respiratory	☐	☐	☐	☐	☐	☐	☐	☐	☐	☐
ICU	☐	☐	☐	☐	☐	☐	☐	☐	☐	☐
Infectious Disease	☐	☐	☐	☐	☐	☐	☐	☐	☐	☐
Cardiology	☐	☐	☐	☐	☐	☐	☐	☐	☐	☐
Neurology	☐	☐	☐	☐	☐	☐	☐	☐	☐	☐
Diabetology	☐	☐	☐	☐	☐	☐	☐	☐	☐	☐
Liaison Psychiatry	☐	☐	☐	☐	☐	☐	☐	☐	☐	☐

19.a. (Continued from grid) Which clinician led the initial consult? Please select all that apply.

- ☐ General Practitioner
- ☐ Nurse (please tick other and state grade)

☐ Other

19.a.i. If you selected Other, please specify specialism, staff group and grade:

20. In wave two (Oct 2020-March 2021) which clinician led the initial consult? Select as appropriate.

	Consultant	Registrar	Staff Grade	Specialist Nurse	Advanced Clinical Practitioner	Physiotherapist	Clinical Psychologists	Occupational Therapists	Dieticians	Speech and Language Therapist
Respiratory	☐	☐	☐	☐	☐	☐	☐	☐	☐	☐
ICU	☐	☐	☐	☐	☐	☐	☐	☐	☐	☐
Infectious Disease	☐	☐	☐	☐	☐	☐	☐	☐	☐	☐
Cardiology	☐	☐	☐	☐	☐	☐	☐	☐	☐	☐
Neurology	☐	☐	☐	☐	☐	☐	☐	☐	☐	☐
Diabetology	☐	☐	☐	☐	☐	☐	☐	☐	☐	☐
Liaison Psychiatry	☐	☐	☐	☐	☐	☐	☐	☐	☐	☐

20.a. (Continued from grid) Which clinician led the initial consult? Please select all that apply.

☐ General Practitioner
☐ Nurse (please tick other and state grade)
☐ Other

20.a.i. If you selected Other, please specify specialism, staff group and grade:

21. In wave one (Feb-Sept 2020) which other clinicians were available in the post-hospitalisation service? Select as appropriate.

	Consultant	Registrar	Staff Grade	Specialist Nurse	Advanced Clinical Practitioner	Physiotherapist	Clinical Psychologists	Occupational Therapists	Dieticians	Speech and Language Therapist
Respiratory	☐	☐	☐	☐	☐	☐	☐	☐	☐	☐
ICU	☐	☐	☐	☐	☐	☐	☐	☐	☐	☐
Infectious Disease	☐	☐	☐	☐	☐	☐	☐	☐	☐	☐
Cardiology	☐	☐	☐	☐	☐	☐	☐	☐	☐	☐

Neurology	☐	☐	☐	☐	☐	☐	☐	☐	☐	☐
Diabetology	☐	☐	☐	☐	☐	☐	☐	☐	☐	☐
Liaison Psychiatry	☐	☐	☐	☐	☐	☐	☐	☐	☐	☐

21.a. (Continued from grid) Which clinicians were available in the post-hospitalisation service? Please select all that apply.

- ☐ General Practitioner
- ☐ Nurse (please tick other and state grade)
- ☐ Clinical Physiologist
- ☐ Other

21.a.i. If you selected Other, please specify specialism, staff group and grade.

22. In wave two (Oct 2020-March 2021) which other clinicians were available in the post-hospitalisation service? Select as appropriate.

	Consultant	Registrar	Staff Grade	Specialist Nurse	Advanced Clinical Practitioner	Physiotherapist	Clinical Psychologists	Occupational Therapists	Dieticians	Speech and Language Therapist
Respiratory	☐	☐	☐	☐	☐	☐	☐	☐	☐	☐
ICU	☐	☐	☐	☐	☐	☐	☐	☐	☐	☐
Infectious Disease	☐	☐	☐	☐	☐	☐	☐	☐	☐	☐
Cardiology	☐	☐	☐	☐	☐	☐	☐	☐	☐	☐
Neurology	☐	☐	☐	☐	☐	☐	☐	☐	☐	☐
Diabetology	☐	☐	☐	☐	☐	☐	☐	☐	☐	☐
Liaison Psychiatry	☐	☐	☐	☐	☐	☐	☐	☐	☐	☐

22.a. (Continued from grid) Which clinicians were available in the post-hospitalisation service? Please select all that apply.

- ☐ General Practitioner
- ☐ Nurse (please tick other and state grade)
- ☐ Clinical Physiologist
- ☐ Other

22.a.i. If you selected Other, please specify specialism, staff group and grade.

23. In wave one (Feb-Sept 2020) did the service include access to a COVID MDT meeting? If no, please select no and progress to question 24.

- ○ Yes
- ○ No

23.a. If yes, which clinicians were part of the MDT meeting? Select as appropriate.

	Consultant	Registrar	Staff Grade	Specialist Nurse	Advanced Clinical Practitioner	Physiotherapist	Clinical Psychologists	Occupational Therapists	Dieticians	Speech and Language Therapist
Respiratory	☐	☐	☐	☐	☐	☐	☐	☐	☐	☐
ICU	☐	☐	☐	☐	☐	☐	☐	☐	☐	☐
Infectious Disease	☐	☐	☐	☐	☐	☐	☐	☐	☐	☐
Cardiology	☐	☐	☐	☐	☐	☐	☐	☐	☐	☐
Neurology	☐	☐	☐	☐	☐	☐	☐	☐	☐	☐
Diabetology	☐	☐	☐	☐	☐	☐	☐	☐	☐	☐
Liaison Psychiatry	☐	☐	☐	☐	☐	☐	☐	☐	☐	☐

23.a.i. (Continued from grid) Which clinicians were part of the MDT meeting? Please select all that apply.

- ☐ General Practitioner
- ☐ Nurse (please tick other and state grade)
- ☐ Radiologist
- ☐ Social Worker
- ☐ Other

23.a.i.a. If you selected Other, please specify:

24. In wave two (Oct 2020-March 2021) did the service include access to a COVID MDT meeting? If no, please select no and progress to question 25.

- ○ Yes
- ○ No

24.a. If yes, which clinicians were part of the MDT meeting? Select as appropriate.

	Consultant	Registrar	Staff Grade	Specialist Nurse	Advanced Clinical Practitioner	Physiotherapist	Clinical Psychologists	Occupational Therapists	Dieticians	Speech and Language Therapist
Respiratory	☐	☐	☐	☐	☐	☐	☐	☐	☐	☐
ICU	☐	☐	☐	☐	☐	☐	☐	☐	☐	☐

Infectious Disease	☐	☐	☐	☐	☐	☐	☐	☐	☐	☐
Cardiology	☐	☐	☐	☐	☐	☐	☐	☐	☐	☐
Neurology	☐	☐	☐	☐	☐	☐	☐	☐	☐	☐
Diabetology	☐	☐	☐	☐	☐	☐	☐	☐	☐	☐
Liaison Psychiatry	☐	☐	☐	☐	☐	☐	☐	☐	☐	☐

24.a.i. (Continued from grid) Which clinicians were part of the MDT meeting? Please select all that apply.

- ☐ General Practitioner
- ☐ Nurse (please tick other and state grade)
- ☐ Radiologist
- ☐ Social Worker
- ☐ Other

24.a.i.a. If you selected Other, please specify:

25. In wave one (Feb-Sept 2020) if there was a MDT meeting were all patients reviewed at the meeting?

○ Yes ○ No ○ Not applicable

25.a. If no, how was the decision made? Please select all that apply.

- ☐ Clinician assessment
- ☐ Test results
- ☐ Other

25.a.i. If you selected Other, please specify:

26. In wave two (Oct 2020-March 2021) if there was a MDT meeting were all patients reviewed at the meeting?

○ Yes ○ No ○ Not applicable

26.a. If no, how was the decision made? Please select all that apply.

- ☐ Clinician assessment

☐ Test results
☐ Other

26.a.i. If you selected Other, please specify:

27. In wave one (Feb-Sept 2020) which investigations were available at the time of the post-hospitalisation service? Please select all that apply.

☐ Phlebotomy
☐ Lung Function
☐ Chest radiography
☐ Other

27.a. If you selected Other, please specify:

28. In wave two (Oct 2020-March 2021) which investigations were available at the time of the post-hospitalisation service? Please select all that apply.

☐ Phlebotomy
☐ Lung Function
☐ Chest radiography
☐ Other

28.a. If you selected Other, please specify:

29. In wave one (Feb-Sept 2020) how was the initial assessment at the post hospitalisation service delivered? Please select all that apply.

☐ Face to face
☐ Telephone
☐ Video call
☐ Other

29.a. If you selected Other, please specify:

30. In wave two (Oct 2020-March 2021) how was the initial assessment at the post hospitalisation service delivered? Please select all that apply.

- ☐ Face to face
- ☐ Telephone
- ☐ Video call
- ☐ Other

30.a. If you selected Other, please specify:

31. In wave one (Feb-Sept 2020) did patients have access to mental health or psychological services?

○ Yes ○ No

31.a. If yes, please select all that apply.

- ☐ Post ICU psychology service
- ☐ Psychiatric liaison service
- ☐ Improving Access to Psychological Therapies (IAPT)
- ☐ Acute Hospital Clinical Health Psychology service
- ☐ Community Mental Health Team (adult)
- ☐ Community Mental Health Team (older adult)
- ☐ Psychological Therapies service for serious mental illness (SMI)
- ☐ Mental health Crisis Resolution and Home Treatment service
- ☐ In patient mental health service
- ☐ Counselling services (Third sector or primary care based)
- ☐ Private providers of psychological therapy
- ☐ Other

31.a.i. If you selected Other, please specify:

32. In wave two (Oct 2020-March 2021) did patients have access to mental health or psychological services?

- Yes
- No

32.a. If yes, please select all that apply.

- Post ICU psychology service
- Psychiatric liaison service
- Improving Access to Psychological Therapies (IAPT)
- Acute Hospital Clinical Health Psychology service
- Community Mental Health Team (adult)
- Community Mental Health Team (older adult)
- Psychological Therapies service for serious mental illness (SMI)
- Mental health Crisis Resolution and Home Treatment service
- In patient mental health service
- Counselling services (Third sector or primary care based)
- Private providers of psychological therapy
- Other

32.a.i. If you selected Other, please specify:

33. In wave one (Feb-Sept 2020) if the post-hospitalisation service did not include an MDT or the MDT did not include a social worker, how was a social care assessment accessed?

- Refer directly
- Ask GP to refer
- No access
- Other

33.a. If you selected Other, please specify:

34. In wave two (Oct 2020-March 2021) if the post-hospitalisation service did not include an MDT or the MDT did not include a social worker, how was a social care assessment accessed?

- Refer directly
- Ask GP to refer
- No access
- Other

34.a. If you selected Other, please specify:

35. In wave one (Feb-Sept 2020) did the post-hospitalisation service team access social prescribing?

- Yes
- No

35.a. If yes, how did the post-hospitalisation team access social prescribing? Please select all that apply.

- Through an OT as part of the MDT
- Through a GP
- No access available
- Other

35.a.i. If you selected Other, please specify:

36. In wave two (Oct 2020-March 2021) did the post-hospitalisation service team access social prescribing?

- Yes
- No

36.a. If yes, how did the post-hospitalisation team access social prescribing? Please select all that apply.

- Through an OT as part of the MDT
- Through a GP
- No access available
- Other

36.a.i. If you selected Other, please specify:

37. Please add any additional information you think may be helpful.

Rehabilitation Questions

We would now like to find out about provision of COVID-19 specfic and routine rehabilitation services for COVID-19 patients.

38. If a different person is completing the rehabilition questions please state the hospital you work in.

39. If a different person is completing the rehabilitation questions what is your role in the post-hospitalisation COVID-19 service?

40. In wave one (Feb-Sept 2020) did patients have access to rehabilitation services?

- Yes
- No

40.a. If yes, was it a COVID specific service?

- Yes
- No

40.a.i. If yes, who was the service delivered by?

- NHS Secondary Care Trust
- NHS Community Provider
- Private Provider
- Other

40.a.i.a. If you selected Other, please specify:

41. In wave one (Feb-Sept 2020) if patients did not have access to a COVID specific rehabilitation service did they have access to existing rehabilitation services?

- Yes
- No
- Not applicable

41.a. If yes, which existing services did they have access to? Please select all that apply.

- ☐ General Practitioner
- ☐ MSK
- ☐ Cardiac rehab
- ☐ Pulmonary rehab
- ☐ Neuro rehab
- ☐ Sports Medicine
- ☐ Chronic Fatigue
- ☐ Clinical Psychologists
- ☐ Other

41.a.i. If you selected Other, please specify:

42. In wave two (Oct 2020-March 2021) did patients have access to rehabilitation services?

○ Yes ○ No

42.a. If yes, was it a COVID specific service?

○ Yes ○ No

42.a.i. If yes, who was the service delivered by?

- ☐ NHS Secondary Care Trust
- ☐ NHS Community Provider
- ☐ Private Provider
- ☐ Other

42.a.i.a. If you selected Other, please specify:

43. In wave two (Oct 2020-March 2021) if patients did not have access to a COVID specific rehabilitation service did they have access to existing rehabilitation services?

○ Yes
○ No

- Not applicable

43.a. If yes, which existing services did they have access to? Please select all that apply.

- General Practitioner
- MSK
- Cardiac rehab
- Pulmonary rehab
- Neuro rehab
- Sports Medicine
- Chronic Fatigue
- Clinical Psychologists
- Other

43.a.i. If you selected Other, please specify:

44. The next section asks questions about the structure and content of COVID-19 specific and existing rehabilitation services. If you have answered **YES** your patients **DO HAVE** access to rehabilitation services (either COVID specific or existing) please select continue to next section. If you answered NO your patients **DO NOT** have access to rehabilitation services please select yes submit.

- Yes submit
- Continue to next section

Rehabilitation Service Structure and Content Questions

45. In wave one (Feb-Oct 2020) was the rehabilitation programme delivered by the same organisation that delivered the post-hospitalisation COVID-19 service?

- ◯ Yes
- ◯ No
- ◯ Other (if more than one organisation please select other to answer)

45.a. If you selected Other, please specify:

46. In wave two (Oct 2020-March 2021) was the rehabilitation programme delivered by same organisation that delivered the post-hospitalisation COVID-19 service?

- ◯ Yes
- ◯ No
- ◯ Other (if more than one organisation please select other to answer)

46.a. If you selected Other, please specify:

47. In wave one (Feb-Sept 2020) which clinicians were involved in the delivery of rehabilitation? Please select all that apply.

- ☐ Doctors
- ☐ Nurses
- ☐ Physiotherapist
- ☐ Occupational Therapist
- ☐ Speech and Language Therapist
- ☐ Dietician
- ☐ Advanced Clinical Practitioner
- ☐ Mental health professionals
- ☐ Other

47.a. If you selected Other, please specify:

48. In wave two (Oct 2020-March 2021) which clinicians were involved in the delivery of rehabilitation? Please select all that apply.

- ☐ Doctors
- ☐ Nurses
- ☐ Physiotherapist
- ☐ Occupational Therapist
- ☐ Speech and Language Therapist
- ☐ Dietician
- ☐ Advanced Clinical Practitioner
- ☐ Mental health professionals
- ☐ Other

48.a. If you selected Other, please specify:

49. In wave one (Feb-Sept 2020) which services were involved in the delivery of rehabilitation? Please select all that apply.

- ☐ General Practice
- ☐ MSK
- ☐ Cardiac rehab
- ☐ Pulmonary rehab
- ☐ Neuro rehab
- ☐ Sports Medicine
- ☐ Chronic Fatigue
- ☐ Clinical Psychologists
- ☐ Other

49.a. If you selected Other, please specify:

50. In wave two (Oct 2020-March 2021) which services were involved in the delivery of rehabilitation? Please select all that apply.

- ☐ General Practice
- ☐ MSK
- ☐ Cardiac rehab
- ☐ Pulmonary rehab
- ☐ Neuro rehab
- ☐ Sports Medicine
- ☐ Chronic Fatigue
- ☐ Clinical Psychologists

- [ ] Other

50.a. If you selected Other, please specify:

51. In wave one (Feb-Sept 2020) what was the mode of delivery for the rehabilitation service? Please select all that apply.

- [ ] Face to face
- [ ] Virtual
- [ ] Your COVID Recovery (Phase 2)
- [ ] Living with Covid Recovery app
- [ ] Other digital
- [ ] Other home based
- [ ] Other

51.a. If you selected Other, please specify:

52. In wave two (Oct 2020-March 2021) what was the mode of delivery for the rehabilitation service? Please select all that apply.

- [ ] Face to face
- [ ] Virtual
- [ ] Your COVID Recovery (Phase 2)
- [ ] Living with Covid Recovery app
- [ ] Other digital
- [ ] Other home based
- [ ] Other

52.a. If you selected Other, please specify:

53. In wave one (Feb-Sept 2020) how was mode of delivery decided. Please select all that apply.

- [ ] Patient preference
- [ ] Severity of symptoms

- ☐ Safety
- ☐ Availability of staff
- ☐ Influence of service pressures
- ☐ Other

53.a. If you selected Other, please specify:

54. In wave two (Oct 2020-March 2021) how was mode of delivery decided? Please select all that apply?

- ☐ Patient preference
- ☐ Severity of symptoms
- ☐ Safety
- ☐ Availability of staff
- ☐ Influence of service pressures.
- ☐ Other

54.a. If you selected Other, please specify:

55. In wave one (Feb-Sept 2020) was the rehabilitation face-to-face?

○ Yes ○ No

55.a. If yes, what was the number of patients per individual class?

56. In wave two (Oct 2020-March 2021) was the the rehabilitation was face-to-face?

☐ Yes ☐ No

56.a. If yes, what was the number of patients per individual class?

57. In wave one (Feb-Sept 2020) what was the duration of the rehabilitation programme? Please state number of weeks.

58. In wave two (Oct 2020-March 2021) what was the duration of the rehabilitation programme? Please state number of weeks.

59. In wave one (Feb-Sept 2020) what was the number of planned supervised sessions in the rehabilitation programme?

60. In wave two (Oct 2020-March 2021) what was the number of planned supervised sessions in the rehabilitation programme?

61. In wave one (Feb-Sept 2020) did the rehabilitation programme include an initial assessment?

- Yes
- No

62. In wave two (Oct 2020-March 2021) did the rehabilitation programme include an initial assessment?

- Yes
- No

63. In wave one (Feb-Sept 2020) did the rehabilitation programme include a discharge assessment?

- Yes
- No

64. In wave two (Oct 2020-March 2021) did the rehabilitation programme include a discharge assessment?

- Yes
- No

65. In wave one (Feb-March 2020) what was included in the rehabilitation programme? Please select all that apply.

- Aerobic exercises
- Strength exercises

- Education
- Self-management information/advice
- Other

65.a. If you selected Other, please specify:

66. In wave two (Oct 2020-March 2021) what was included in the rehabilitation programme? Please select all that apply.

- Aerobic exercises
- Strength exercises
- Education
- Self-management information/advice
- Other

66.a. If you selected Other, please specify:

67. In wave one (Feb-Sept 2020) were exercise outcome measures used?

- Yes
- No

67.a. If yes, which exercise outcome measures were used? Please select all that apply.

- Exercise capacity
- Incremental Shuttle Walking Test (ISWT)
- Endurance Shuttle Walking Test (ESWT)
- 6 Minute Walking Distance (6MWD)
- Other

67.a.i. If you selected Other, please specify:

68. In wave two (Oct 2020-March 2021) were exercise outcomes used?

- ○ Yes
- ○ No

68.a. If yes, which outcome measures were used?

- ☐ Exercise capacity
- ☐ Incremental Shuttle Walking Test (ISWT)
- ☐ Endurance Shuttle Walking Test (ESWT)
- ☐ 6 Minute Walking Distance (6MWD)
- ☐ Other

68.a.i. If you selected Other, please specify:

69. In wave one (Feb-Sept 2020) were generic health questionnaires used?

- ○ Yes
- ○ No

69.a. If yes, which generic If yes, which generic health questionnaires were used?

- ☐ Euro Qol 5 domain (EQ5D)
- ☐ SF-36
- ☐ Other

69.a.i. If you selected Other, please specify:

70. In wave two (Oct 2020-March 2021) were generic health questionnaires used?

- ○ Yes
- ○ No

70.a. If yes, which generic health questionnaires used?

- ☐ Euro Qol 5 domain (EQ5D)
- ☐ SF-36
- ☐ Other

70.a.i. If you selected Other, please specify:

71. In wave one (Feb-Sept 2020) were symptoms measured?

- Yes
- No

71.a. If yes, which symptoms were measured? Please select all that apply.

- Fatigue
- Breathlessness
- Pain
- Cognition
- Other

71.a.i. If you selected Other, please specify:

72. In wave two (Oct 2020-March 2021) were symptoms measured?

- Yes
- No

72.a. If yes, which symptoms were measured? Please select all that apply.

- Fatigue
- Breathlessness
- Pain
- Cognition
- Other

72.a.i. If you selected Other, please specify:

73. In wave one (Feb-Sept 2020) were functional outcome measures used?

- Yes
- No

73.a. If yes, which functional outcome measures were used?

- ☐ Sit to stand
- ☐ Other

73.a.i. If you selected Other, please specify:

74. In wave two (Oct 2020-March 2021) were functional outcome measures used?

- ○ Yes
- ○ No

74.a. If yes, which functional outcome measures used?

- ☐ Sit to stand
- ☐ Other

74.a.i. If you selected Other, please specify:

75. In wave one (Feb-Sept 2020) were strength outcome measures used?

- ○ Yes
- ○ No

75.a. If yes, which strength outcome measures were used? Please select all that apply.

- ☐ Handgrip
- ☐ Quadriceps maximal voluntary contraction force (QMVC)
- ☐ Other

75.a.i. If you selected Other, please specify:

76. In wave two (Oct 2020-Marach 2021) were strength outcome measures used?

- ○ Yes
- ○ No

76.a. If yes, which strength outcome measures used?

- ☐ Handgrip
- ☐ Quadriceps maximal voluntary contraction force (QMVC)
- ☐ Other

76.a.i. If you selected Other, please specify:

77. In wave one (Feb-Sept 2020) were emotional state outcome measures used?

- ○ Yes
- ○ No

77.a. If yes, which emotional state outcome measures were used. Please select all that apply.

- ☐ Patient Health Questionnaire (PHQ)
- ☐ Generalised Anxiety Disorder Assessment (GADS)
- ☐ Hospital Anxiety and Depression Scale (HADS)
- ☐ Other

77.a.i. If you selected Other, please specify:

78. In wave two (Oct 2020-March 2021) were emotional state outcome measures used?

- ○ Yes
- ○ No

78.a. If yes, which emotional state outcomes were used? Please select all that apply.

- ☐ Patient Health Questionnaire (PHQ)
- ☐ Generalised Anxiety Disorder Assessment (GADS)
- ☐ Hospital Anxiety and Depression Scale (HADS)
- ☐ Other

78.a.i. If you selected Other, please specify:

79. Please add any additional information you think may be helpful.

Check page

80. If you are finished, click submit. once you finish this page, the survey will submit

- ○ Submit

Final page

Thank you for completing the survey.

PHOSP Mapping Service Survey - Rehab Questions

Post-Hospitalisation COVID-19 Follow-up Service Mapping Survey - Rehabilitation Questions

Introduction

The following questions are related to the structure and content of COVID-19 specific and existing rehabilitation services.

You may need assistance of the rehabilitation team to complete the questions.

Please note: only one person can complete the survey at a time.

If you have any questions please contact Dr Charlotte Overton, Health Service Researcher, via email - charlotte.overton@leicester.ac.uk

Rehabilitation Service Structure and Content Questions

1. What is the name of the hospital you work in?

2. What is your role the in the post-hospitalisation COVID-19 service?

3. In wave one (Feb-Oct 2020) was the rehabilitation programme delivered by the same organisation that delivered the post-hospitalisation COVID-19 service?

- Yes
- No
- Other (if more than one organisation please select other to answer)

3.a. If you selected Other, please specify:

4. In wave two (Oct 2020-March 2021) was the rehabilitation programme delivered by same organisation that delivered the post-hospitalisation COVID-19 service?

- Yes
- No

- Other (if more than one organisation please select other to answer)

4.a. If you selected Other, please specify:

[ ]

5. In wave one (Feb-Sept 2020) which clinicians were involved in the delivery of rehabilitation? Please select all that apply.

- [ ] Doctors
- [ ] Nurses
- [ ] Physiotherapist
- [ ] Occupational Therapist
- [ ] Speech and Language Therapist
- [ ] Dietician
- [ ] Advanced Clinical Practitioner
- [ ] Mental health professionals
- [ ] Other

5.a. If you selected Other, please specify:

[ ]

6. In wave two (Oct 2020-March 2021) which clinicians were involved in the delivery of rehabilitation? Please select all that apply.

- [ ] Doctors
- [ ] Nurses
- [ ] Physiotherapist
- [ ] Occupational Therapist
- [ ] Speech and Language Therapist
- [ ] Dietician
- [ ] Advanced Clinical Practitioner
- [ ] Mental health professionals
- [ ] Other

6.a. If you selected Other, please specify:

7. In wave one (Feb-Sept 2020) which services were involved in the delivery of rehabilitation? Please select all that apply.

- [ ] General Practice
- [ ] MSK
- [ ] Cardiac rehab
- [ ] Pulmonary rehab
- [ ] Neuro rehab
- [ ] Sports Medicine
- [ ] Chronic Fatigue
- [ ] Clinical Psychologists
- [ ] Other

7.a. If you selected Other, please specify:

8. In wave two (Oct 2020-March 2021) which services were involved in the delivery of rehabilitation? Please select all that apply.

- ☐ General Practice
- ☐ MSK
- ☐ Cardiac rehab
- ☐ Pulmonary rehab
- ☐ Neuro rehab
- ☐ Sports Medicine
- ☐ Chronic Fatigue
- ☐ Clinical Psychologists
- ☐ Other

8.a. If you selected Other, please specify:

9. In wave one (Feb-Sept 2020) what was the mode of delivery for the rehabilitation service? Please select all that apply.

- ☐ Face to face
- ☐ Virtual
- ☐ Your COVID Recovery (Phase 2)

- [ ] Living with Covid Recovery app
- [ ] Other digital
- [ ] Other home based
- [ ] Other

9.a. If you selected Other, please specify:

10. In wave two (Oct 2020-March 2021) what was the mode of delivery for the rehabilitation service? Please select all that apply.

- [ ] Face to face
- [ ] Virtual
- [ ] Your COVID Recovery (Phase 2)
- [ ] Living with Covid Recovery app
- [ ] Other digital
- [ ] Other home based
- [ ] Other

10.a. If you selected Other, please specify:

11. In wave one (Feb-Sept 2020) how was mode of delivery decided. Please select all

that apply.

- Patient preference
- Severity of symptoms
- Safety
- Availability of staff
- Influence of service pressures
- Other

11.a. If you selected Other, please specify:

12. In wave two (Oct 2020-March 2021) how was mode of delivery decided? Please select all that apply?

- Patient preference
- Severity of symptoms
- Safety
- Availability of staff
- Influence of service pressures.
- Other

12.a. If you selected Other, please specify:

13. In wave one (Feb-Sept 2020) was the rehabilitation face-to-face?

- ○ Yes
- ○ No

13.a. If yes, what was the number of patients per individual class?

[ ]

14. In wave two (Oct 2020-March 2021) was the the rehabilitation was face-to-face?

- ☐ Yes
- ☐ No

14.a. If yes, what was the number of patients per individual class?

[ ]

15. In wave one (Feb-Sept 2020) what was the duration of the rehabilitation programme? Please state number of weeks.

[ ]

16. In wave two (Oct 2020-March 2021) what was the duration of the rehabilitation programme? Please state number of weeks.

[ ]

17. In wave one (Feb-Sept 2020) what was the number of planned supervised sessions in the rehabilitation programme?

18. In wave two (Oct 2020-March 2021) what was the number of planned supervised sessions in the rehabilitation programme?

19. In wave one (Feb-Sept 2020) did the rehabilitation programme include an initial assessment?

- Yes
- No

20. In wave two (Oct 2020-March 2021) did the rehabilitation programme include an initial assessment?

- Yes
- No

21. In wave one (Feb-Sept 2020) did the rehabilitation programme include a discharge assessment?

- Yes
- No

22. In wave two (Oct 2020-March 2021) did the rehabilitation programme include a discharge assessment?

- ○ Yes
- ○ No

23. In wave one (Feb-March 2020) what was included in the rehabilitation programme? Please select all that apply.

- ☐ Aerobic exercises
- ☐ Strength exercises
- ☐ Education
- ☐ Self-management information/advice
- ☐ Other

23.a. If you selected Other, please specify:

24. In wave two (Oct 2020-March 2021) what was included in the rehabilitation programme? Please select all that apply.

- ☐ Aerobic exercises
- ☐ Strength exercises
- ☐ Education
- ☐ Self-management information/advice
- ☐ Other

24.a. If you selected Other, please specify:

25. In wave one (Feb-Sept 2020) were exercise outcome measures used?

- Yes
- No

25.a. If yes, which exercise outcome measures were used? Please select all that apply.

- Exercise capacity
- Incremental Shuttle Walking Test (ISWT)
- Endurance Shuttle Walking Test (ESWT)
- 6 Minute Walking Distance (6MWD)
- Other

25.a.i. If you selected Other, please specify:

26. In wave two (Oct 2020-March 2021) were exercise outcomes used?

- Yes
- No

26.a. If yes, which outcome measures were used?

- ☐ Exercise capacity
- ☐ Incremental Shuttle Walking Test (ISWT)
- ☐ Endurance Shuttle Walking Test (ESWT)
- ☐ 6 Minute Walking Distance (6MWD)
- ☐ Other

26.a.i. If you selected Other, please specify:

27. In wave one (Feb-Sept 2020) were generic health questionnaires used?

○ Yes ○ No

27.a. If yes, which generic If yes, which generic health questionnaires were used?

- ☐ Euro Qol 5 domain (EQ5D)
- ☐ SF-36
- ☐ Other

27.a.i. If you selected Other, please specify:

28. In wave two (Oct 2020-March 2021) were generic health questionnaires used?

- ○ Yes
- ○ No

28.a. If yes, which generic health questionnaires used?

- ☐ Euro Qol 5 domain (EQ5D)
- ☐ SF-36
- ☐ Other

28.a.i. If you selected Other, please specify:

29. In wave one (Feb-Sept 2020) were symptoms measured?

- ○ Yes
- ○ No

29.a. If yes, which symptoms were measured? Please select all that apply.

- ☐ Fatigue
- ☐ Breathlessness
- ☐ Pain
- ☐ Cognition
- ☐ Other

29.a.i. If you selected Other, please specify:

30. In wave two (Oct 2020-March 2021) were symptoms measured?

- Yes
- No

30.a. If yes, which symptoms were measured? Please select all that apply.

- Fatigue
- Breathlessness
- Pain
- Cognition
- Other

30.a.i. If you selected Other, please specify:

31. In wave one (Feb-Sept 2020) were functional outcome measures used?

- Yes
- No

31.a. If yes, which functional outcome measures were used?

- Sit to stand

☐ Other

31.a.i. If you selected Other, please specify:

32. In wave two (Oct 2020-March 2021) were functional outcome measures used?

○ Yes ○ No

32.a. If yes, which functional outcome measures used?

☐ Sit to stand
☐ Other

32.a.i. If you selected Other, please specify:

33. In wave one (Feb-Sept 2020) were strength outcome measures used?

○ Yes ○ No

33.a. If yes, which strength outcome measures were used? Please select all that apply.

- [ ] Handgrip
- [ ] Quadriceps maximal voluntary contraction force (QMVC)
- [ ] Other

33.a.i. If you selected Other, please specify:

34. In wave two (Oct 2020-Marach 2021) were strength outcome measures used?

- ○ Yes
- ○ No

34.a. If yes, which strength outcome measures used?

- [ ] Handgrip
- [ ] Quadriceps maximal voluntary contraction force (QMVC)
- [ ] Other

34.a.i. If you selected Other, please specify:

35. In wave one (Feb-Sept 2020) were emotional state outcome measures used?

- ○ Yes
- ○ No

35.a. If yes, which emotional state outcome measures were used. Please select all that apply.

- [ ] Patient Health Questionnaire (PHQ)
- [ ] Generalised Anxiety Disorder Assessment (GADS)
- [ ] Hospital Anxiety and Depression Scale (HADS)
- [ ] Other

35.a.i. If you selected Other, please specify:

36. In wave two (Oct 2020-March 2021) were emotional state outcome measures used?

- ( ) Yes
- ( ) No

36.a. If yes, which emotional state outcomes were used? Please select all that apply.

- [ ] Patient Health Questionnaire (PHQ)
- [ ] Generalised Anxiety Disorder Assessment (GADS)
- [ ] Hospital Anxiety and Depression Scale (HADS)
- [ ] Other

36.a.i. If you selected Other, please specify:

37. Please add any additional information you think may be helpful.

Check page

38. If you are finished, click submit. once you finish this page, the survey will submit

- ○ Submit

Final page

Thank you for completing the survey.
