# Supplementary material for: A typology of healthcare pathways after hospital discharge for adults with COVID-19: the evolution of UK services during pandemic conditions
Source: ERJ Open Res. 2023 Aug 14;9(4):00565-2022. doi: 10.1183/23120541.00565-2022 (PMC10423987; doi:10.1183/23120541.00565-2022)

Appendices: Tables

Table S1. Agenda for virtual stakeholder event.

**2.00pm - 2.05pm**	**Welcome and Introduction**	Professor Andy Briggs
**2.05pm – 2.15pm**	**Update on NHS England Long Covid plan**	Cathy Hassell
**2.15pm – 2.20pm**	**Overview of PHOSP COVID study**	Dr Rachael Evans
**2.20pm – 2.30pm**	**Overview of PHOSP COVID – Health Services Research project**	Professor Andy Briggs
**2.30pm – 2.40pm**	**Survey results and classification of post-hospitalisation pathways**	Dr Rachael Evans and Dr Linzy Houchen-Wolloff
	**Comfort Break**	
**2.45pm – 3.45pm**	**Facilitated discussion**	Dr Rachael Evans, Dr Linzy Houchen-Wolloff and Dr Charlotte Overton

S2. Staffing for post-hospitalisation clinics in both waves

	Wave 1 N (%)	Wave 2 N (%)
**Redeployed**	13 (35%)	12 (33%)
**Permanent**	12 (33%)	13 (36%)
**Temporary**	4 (10%)	6 (16%)
**Other***	8 (21%)	6 (16%)

*** Other: volunteers, where there was capacity-teams helped, research fellows, registrars took it in turns.**

Table S3. Investigations available at the time of the post-hospitalisation service

	Respondents with tests available. N (%) Wave 2 n=37
Phlebotomy, Lung Function, Chest radiography	14 (38%)
Phlebotomy, Lung Function, Chest radiography, Other	9 (24%)
Phlebotomy, Chest radiography	9 (24%)
Chest radiography alone	3 (8%)
Phlebotomy, Chest radiography, Other	2 (6%)
Phlebotomy alone	0 (0%)

Table S4. Staffing and Specialty leading consultations

Speciality Leading	Respondents. N (%)<br>Wave 2 n=37
Respiratory	35 (95%)
ICU	18 (49%)
Other	12 (32%)
Infectious Disease	9 (24%)
Cardiology	5 (14%)
Neurology	3 (8%)
Diabetology	3 (8%)
Liaison Psychiatry	2 (6%)

Other specialties available	Respondents. N (%)<br>Wave 2 n=37
Respiratory	30 (81%)
ICU	18 (49%)
Other	17 (46%)
Infectious Disease	7 (19%)

Cardiology	6 (16%)
Neurology	4 (11%)
Diabetology	4 (11%)
Liaison Psychiatry	4 (11%)

Legend: ***ICU: Intensive Care Unit.***

Table S5. Staffing and Specialty involved in the MDT meetings, data presented as number and (Percentages/ n=19 with MDT clinics)

	Respiratory	ICU	Infectious Disease	Cardiology	Neurology	Diabetology	Liaison Psychiatry
Consultant	16 (84%)	11 (58%)	9 (47%)	6 (32%)	5 (26%)	1 (5%)	4 (21%)
SpR	5 (26%)	2 (11%)	1 (5%)	1 (5%)	0	1 (5%)	0
Specialist Nurse	3 (16%)	4 (21%)	1 (5%)	0	0	0	0
ACP	1 (5%)	0	0	0	0	0	1 (5%)
Physio	9 (47%)	8 (42%)	0	0	0	0	0
Psych	1 (5%)	4 (21%)	1 (5%)	0	0	1 (5%)	1 (5%)
OT	1 (5%)	5 (26%)	0	0	0	0	0
Dietician	1 (5%)	3 (16%)	0	0	0	1 (5%)	1 (5%)
SALT	0	3 (16%)	0	0	0	0	0

Legend: *ICU: Intensive Care Unit, SpR: specialist registrar, ACP: advanced care practitioner, Physio: physiotherapist, Psych: Clinical Psychologists, OT: occupational therapist, SALT: speech and language therapist*

Appendices: Figures

Figure S1. Health Service Assessment Algorithm. Endpoints (the quadrants) are highlighted using dotted borders. Quadrant 1 = Low intensity and pre-specified subgroup of patients, Quadrant 2 = Low intensity and all patients, Quadrant 3 = High intensity and pre-specified subgroup of patients, Quadrant 4 = High intensity and all patients.

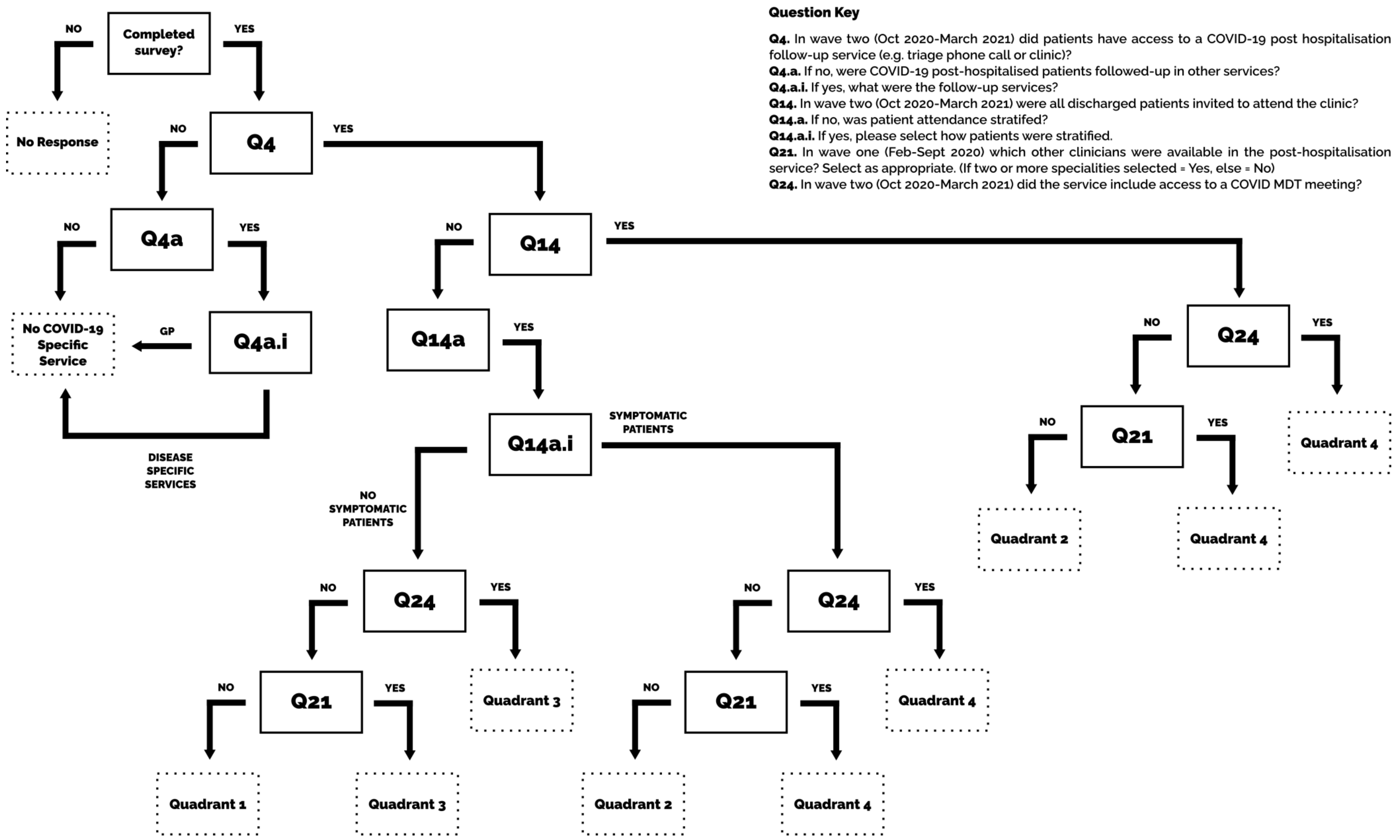

Figure S2. The initial classification of health care pathways produced at the virtual consensus event.

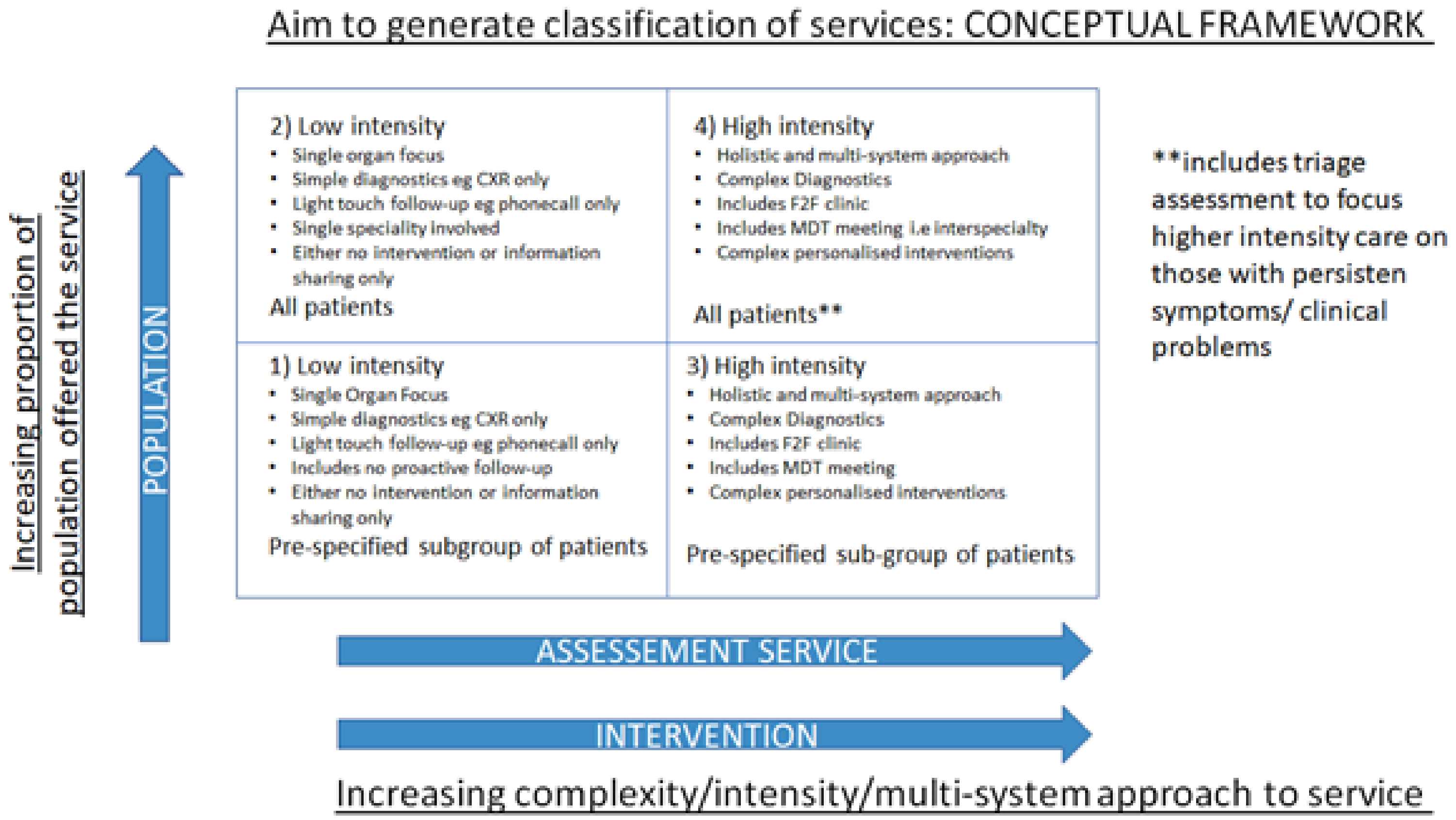

Supplement: Supplementary file 2 [file 00565-2022.supplementary_tables.pdf]
